# Predicting the Distribution of *Sclerodermus sichuanensis* (Hymenoptera: Bethylidae) under Climate Change in China

**DOI:** 10.3390/insects14050475

**Published:** 2023-05-18

**Authors:** Hui Gao, Qianqian Qian, Lijuan Liu, Danping Xu

**Affiliations:** 1 College of Environmental Science and Engineering, China West Normal University, Nanchong 637002, China; hubeihuige@cwnu.edu.cn; 2 College of Life Science, China West Normal University, Nanchong 637002, China; qianqqqchen@foxmail.com; 3 Nanchong Gaoping District Urban and Rural Construction Bureau, Nanchong 637002, China; liu-lj@foxmail.com

**Keywords:** *Sclerodermus sichuanensis*, MaxEnt, climate change, environmental variables, biocontrol

## Abstract

**Simple Summary:**

*Sclerodermus sichuanensis* is a natural enemy of the longicorn beetle and has biological control value, and its habitat area was studied. In this work, we simulated the current distribution of *S. sichuanensis* in China using the Maxent model and ArcGIS software and predicted its distribution in different future periods. The results showed that the Mean Diurnal Range (bio2), Min Temperature of Coldest Month (bio6), Precision of Warmest Quarter (bio18), and Max Temperature of Warmest Month (bio5) were the key environmental variables affecting the distribution of *S. sichuanensis*. Southwest China, represented by Sichuan, Chongqing, Guizhou, and part of North China, are the main concentrations of the current high suitability areas of *S. sichuanensis*. The moderately suitable areas are concentrated in South China and Central China. Most of the high and middle appropriate areas belong to subtropical monsoon climate and temperate monsoon climate. In one of the future periods, the suitable area will be significantly expanded to North and Northwest China, with a total area increase of 81,295 km^2^. This study provides an essential reference for future research on *S. sichuanensis* and the application of forestry pest control.

**Abstract:**

*Sclerodermus sichuanensis* is the natural enemy of the longicorn beetle due to its strong attack ability and high parasitic rate. Its good resistance and fecundity make it have significant biological control value. The Maxent model and ArcGIS software were used to simulate the current distribution of *S. sichuanensis* in China by combining the known distribution information and environmental variables and predict the suitable area of the 2050s (2041–2060) and 2090s (2081–2000) under three climate scenarios (SSP1-2.6, SSP2-4.5. and SSP5-8.5). The results showed that the Mean Diurnal Range (bio2), Min Temperature of the Coldest Month (bio6), Precipitation of the Warmest Quarter (bio18), and Max Temperature of the Warmest Month (bio5) were the key environmental variables affecting the distribution of *S. sichuanensis*. Southwest China and part of North China are the main concentrations of the current high-suitability areas of *S. sichuanensis*. The moderately suitable areas are concentrated in South China and Central China. Under the SSP5-8.5 scenario, the suitable area predicted in the 2050s will expand significantly to North China and Northwest China, with a total increase of 81,295 km^2^. This work provides an essential reference for future research on *S. sichuanensis* and the application of forestry pest control.

## 1. Introduction

*Sclerodermus sichuanensis* is a new parasitic wasp discovered in Luxian County, Sichuan Province 1994 [1]. It belongs to the family Bethylidae (Hymenoptera). *Sclerodermus sichuanensis* has many host species and was initially found parasitizing larvae and pupae of *Semanotus sinoauster*. *Semanotus sinoauster* belongs to the family Cerambycidae of the order Coleoptera. It infests *Cunninghamia lanceolata,* and *Cryptomeria japonica* is a stem-scavenging pest. In addition, it can also parasitize on borer pests such as *Callidium villosulum*, *Semanotus bifasciatus*, *Anoplophora chinensis*, *Monochamus alternatus* [2,3], as well as the latest research on borers such as *Clytus validus*, *Apriona germari*, etc. [4]. *Sclerodermus sichuanensis* has an effective control effect on many species of longicorn beetles and has become the natural enemy of longicorn beetles [5]. Compared with other parasitic wasps, *S. sichuanensis* has stronger searching and attacking ability [6], stronger resistance and fecundity, higher parasitic rate [7], long life span, and short development cycle make it have significant biological control value. Now, after systematic research on *S. sichuanensis* behavior, bioecology, indoor large-scale breeding technology has been further established and widely used to control borers of urban garden trees and economic forests [8].

The spatial distribution area of species is an important ecological and evolutionary feature of species [9], and an essential prerequisite for mastering and using species [10]. At present, the limitations of conventional field survey methods limit our grasp of species distribution. Therefore, a niche model based on niche theory emerged as the time required and has become a new research field in species distribution [11]. The niche model predicts according to the known geographic distribution area of the species and the environmental data of the location [12,13]. The predicted area that meets the niche needs of the species is part of the potential species distribution area in the future. Common niche models include the Climate Extremes Index (CLIMEX), Genetic Algorithm for Rule-set Production (GARP), Ecological Niche Factor Analysis (ENFA), Bioclimate Model (BIOCLIM), Maximum Entropy Method (Maxent), etc. [14]. The MaxEnt model is a machine-learning method based on the maximum entropy principle [15]. Compared with other models, the MaxEnt model can predict species’ closest uniform potential distribution with less sample size information, with accurate prediction, simple operation, and stable performance. Elith et al. (2006) compared the prediction abilities of various niche models and concluded that MaxEnt had the highest prediction ability among 16 models. MaxEnt has been widely used in the potential distribution of species [16], the risk evaluation of species invasion, invasion biology, conservation biology and agronomy [17]. Therefore, the MaxEnt model is selected in this work to predict the distribution area of *S. sichuanensis* [5].

By combining the prediction results of the Geographic Information System (GIS) and MaxEnt model, the weight of each environmental factor affecting the expected distribution of species can be described [18], thereby screening the dominant environmental parameters [19,20]. In this study, climatic variables and elevation were selected. The suitable distribution areas of *S. sichuanensis* in China were analyzed by the MaxEnt model combined with GIS and SPSS statistical analysis software. Key environmental variables affecting the distribution of the parasitic wasp were identified by the Pearson correlation coefficient and the jackknife test. This work could play a role in monitoring the occurrence of *S. sichuanensis* and could be better utilized for biological control. 

## 2. Materials and Methods

### 2.1. Species Data Sources and Processing

**Figure 1:**
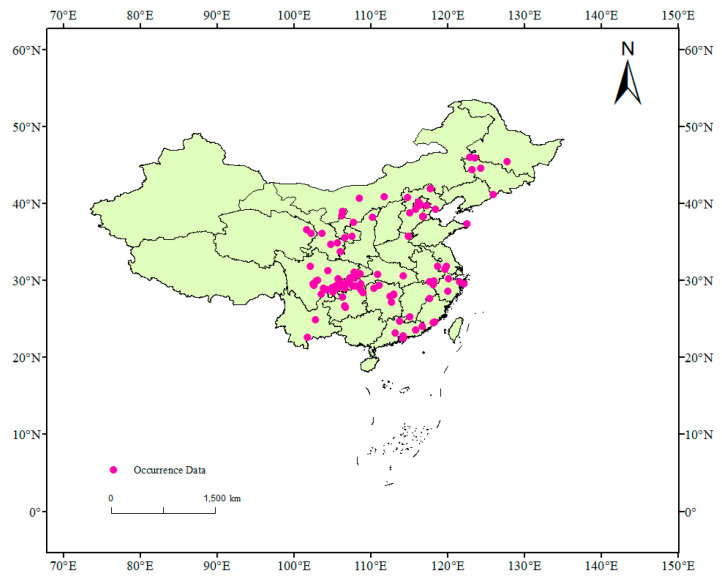
Geographical distribution points of *Sclerodermus sichuanensis* in China.

In this research, the occurrence data were obtained by reviewing relevant literature and field survey data. Finally, the specific latitude and longitude data of distribution sites were confirmed by Google Earth online (http://www.earthol.com/, accessed on 12 January 2023). Each distribution point may cause an overfitting simulation because of a large spatial correlation. To eliminate this impact, the buffer analysis method was used to screen the obtained points in ArcGIS 3.0 software [21]. The spatial resolution of the environmental data used in this study is 2.5 arc minutes (about 4.5 km), and the buffer radius is set to 1.5 km. The buffer area will overlap when two distribution points are in the same buffer zone. At this time, only one distribution point is reserved in the two points. Finally, 121 effective distribution points of *S. sichuanensis* were obtained (Figure 1). These occurrence data have been published on Figshare (see Data Availability Statement), and they come from published information and original findings of the authors. Later, the data was put into Excel tables in specifications, longitude, and latitude and finally generated into CSV format files that Maxent software can support.

### 2.2. Environmental Factors

The distribution of species and their dynamics over time is determined by the characteristics of species and environmental changes, so environmental variables are significant to the ecological distribution of species [22]. To explain the distribution of *S. sichuanensis* more comprehensively, 19 bioclimatic variables and one topographic factor variable were added to the model analysis. All variables were downloaded from the Worldclim database (version 2.0, http://www.worldclim.org/, accessed on 20 January 2023). See Table 1 for specific variables. The current climate data is the average representative value of 1970–2000. The data was downloaded with a precision of 2.5 arc minutes and converted into ASCII format. CMIP6 is the latest climate model available, so it was used for data processing in this study. The United Nations Intergovernmental Panel on Climate Change (IPCC) released its Fifth Assessment Report in 2014, which proposed Shared Socioeconomic Pathways (SSPs). We selected SSP1-2.6, SSP2-4.5, and SSP5-8.5 to represent low, medium, and high SSP scenarios, respectively, to simulate the distribution of suitable habitats in the 2050s (2041–2060) and 2090s (2081–2000).

Since the 19 selected climate variables reflect the characteristics of precipitation and temperature, there will be a certain degree of autocorrelation between these variables, and some environmental variables with low contribution rates need to be further screened out. When establishing the model, the jackknife test was used to determine the contribution rate of each environment variable. Finally, environment variables with a contribution rate greater than 1.0% were retained, and 12 key environment variables were reserved for subsequent model prediction. SPSS 22 software was employed to analyze the Pearson correlation coefficient (*r*). Similarly, to improve model prediction accuracy, only |*r*| < 0.8 variables were retained in the r coefficient. |*r*| ≥ 0.8 indicates a significant correlation between the two variables. At this time, environmental variables that have less impact on species were removed [23].

### 2.3. MaxEnt Modeling

The environmental factors retained after screening and the determined distribution point data were imported into MaxEnt 3.4.4 software to build the distribution model of different habitat suitability for *S. sichuanensis* at present and in the future. 75% of the *S. sichuanensis* records were randomly selected as the training data set to establish the prediction model, and the remaining 25% of the data were treated as the test set to verify the model’s accuracy. To avoid the instability caused by randomly selected data, the initial operation of the model was repeated ten times. The jackknife test was chosen to evaluate the relative importance of variables. The default parameters were RM = 1, FC = LQHPT, and the output form was Logistic. In R software 4.3.0, by calling the ENMeval package to optimize the MaxEnt model [24,25] two parameters of regularization multiplier (RM) and feature classes (FC) were reset. AUC_DIFF_ (the difference between training set AUC and test set AUC) and test omission rate were employed to test the model’s fit to species distribution. The optimal parameters obtained after optimization were adopted to simulate and predict the suitable habitat. The accuracy of the MaxEnt model was tested by the area under the curve (AUC) of the ROC. Generally, the AUC value is between [0, 1]. The closer the value is to 1, the more accurate the prediction result is. As a result, the average training set AUC and the average test set AUC obtained after ten tests were selected to represent the prediction results. AUC between 0.70 and 0.80 indicates that the prediction results are general; 0.81~0.90 indicates that the prediction result is accurate; 0.91~1.00 indicates that the prediction result is more accurate.

We imported the output MaxEnt model results in logistic format into ArcGIS software for data extraction and analyzed the climate suitability. First, the distribution values were divided according to the division method of assessing probability in the IPCC report. Then, habitat suitability was divided into four classes, which were represented by different colors: unsuitable zone (*p* < 0.05, white), low suitability zone (0.05 < *p* < 0.33, yellow), medium suitability zone (0.33 < *p* < 0.66, orange) and high suitability zone (0.66 < *p* < 1, red).

## 3. Results

### 3.1. Model Optimization Results and Accuracy Evaluation

Based on the key environmental variables, the MaxEnt model was built to obtain the ROC curve, which shows that the average AUC value is 0.865, meaning that the model prediction results reach an accurate standard. This shows that the model has a very good prediction result and high reliability for the potentially suitable area of *S. sichuanensis* in China.

### 3.2. Model Performance and Key Environment Variables

After analyzing all climate variables, there are 12 environmental variables whose contribution rate is greater than 1.0% (Table 2). Table 2 shows these key environmental variables’ contribution rate and ranking importance. In descending order, it is Precipitation of Warmest Quarter (bio18), Temperature Seasonality (bio4), Precipitation Seasonality (Coefficient of Variation) (bio15), Precipitation of Wettest Month (bio13), Isothermality (bio3), Min Temperature of Coldest Month (bio6), Precipitation of Coldest Quarter (bio19), Mean Diurnal Range (bio2), Max Temperature of Warmest Month (bio5), Temperature Annual Range (bio7), Mean Temperature of Coldest Quarter (bio11), and Mean Temperature of Wettest Quarter (bio8). The highest contribution rate is 47.7%, bio18, and the second is 17.7% (bio4). The cumulative contribution rate of these 12 key variables is 97.3%, indicating that these variables are essential variables to simulate the future distribution of *S. sichuanensis*.

**Figure 2:**
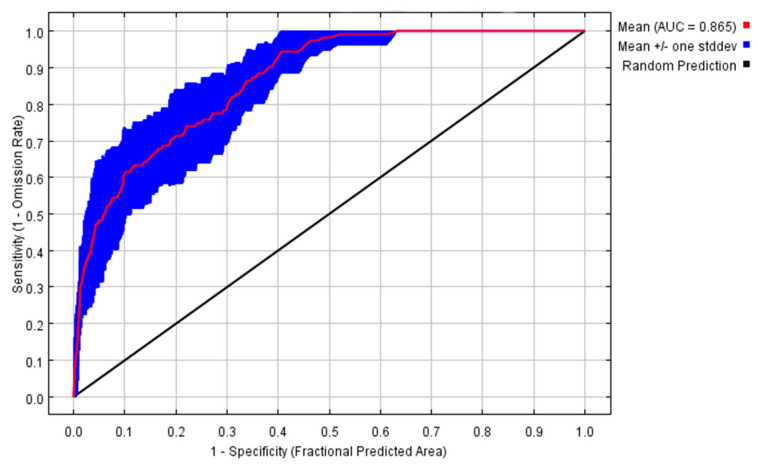
ROC curve of potential distribution prediction of *Sclerodermus sichuanensis*.

Pearson correlation coefficient analysis is conducted for 12 key variables. Finally, nine key environmental factors with |r| < 0.8 were selected to build the MaxEnt model, including bio15, bio18, bio19, bio2, bio3, bio4, bio5, bio6 and bio8. Figure 2 shows the ROC curve of the model, and the average AUC value is 0.865, indicating that the model’s potential distribution prediction results for *S. sichuanensis* are accurate.

### 3.3. Predicting the Current Distribution of Sclerodermus sichuanensis in China

**Figure 3:**
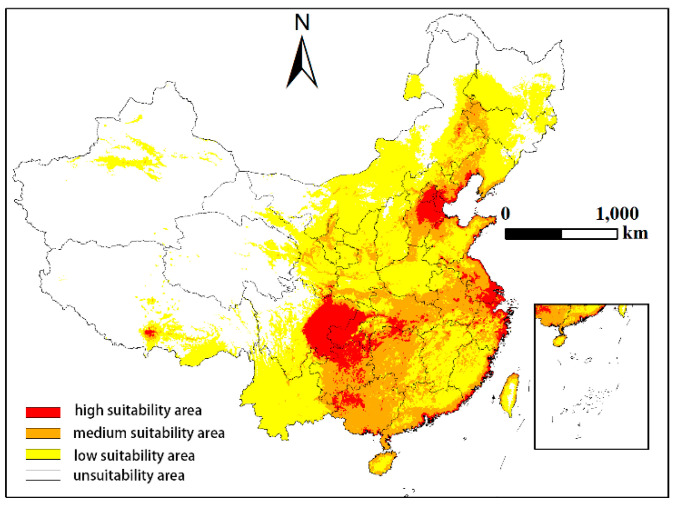
Current suitable climatic distribution of *Sclerodermus sichuanensis* in China. The probability of *S. sichuanensis* is shown by the colour scale in the area. For example, red is a highly suitable area with a probability of higher than 0.66; Orange is a moderately suitable area with a probability of 0.33–0.66; Yellow is a poorly suitable area ranging from 0.05–0.33; White is an unsuitable area.

Considering the key environmental variables and geographic data of *S. sichuanensis* distribution, Maxent model simulation results show that *S. sichuanensis* are distributed in high suitability areas, medium suitability areas, low suitability areas, and unsuitable areas in China (Figure 3). The figure shows that the highly suitable areas for *S. sichuanensis* in China are mainly in plains and basins areas such as Sichuan, Chongqing, Guizhou, Hebei, and Jiangsu, and the climate is concentrated in subtropical monsoon climate and temperate continental monsoon climate. The distribution area of medium and low suitability areas is wider than that of high suitability areas, and they are mainly concentrated in North China, South China, the middle and lower reaches of the Yangtze River, and most of the Northeast. The climate belongs to subtropical monsoon climate and temperate monsoon climate. According to the data in Table 3, the current high suitability area of *S. sichuanensis* is 28,640 km^2^, accounting for 0.3% of the country’s total area. The regions with the largest high suitability area are Sichuan, Chongqing, and Guizhou, accounting for 24.7%, 11.8%, and 11.3% of the high suitability area of the country, respectively. The total area of medium suitable areas is 77,841 m^2^, accounting for 1.8% of the country’s total area. The Guangxi, Hunan, and Hubei provinces have the largest area distribution. The total area of the three provinces is 24,608 m^2^, accounting for 31.6% of the total area of medium-suitable areas of the country. The total area of low suitability is 170,515 m^2^, accounting for 0.8% of the country’s total area. Inner Mongolia, Yunnan, and Heilongjiang have the largest area distribution. The total area of the three provinces is 63,990 m^2^, accounting for 37.5% of the country’s total area of low suitability. In addition, *S. sichuanensis* had the highest usefulness in Hong Kong and Tianjin, with 100% and 99.3% of the total area of each province in the two high suitability zones, respectively. 

### 3.4. Potential Distribution of S. sichuanensis in the Future Period

**Figure 4:**
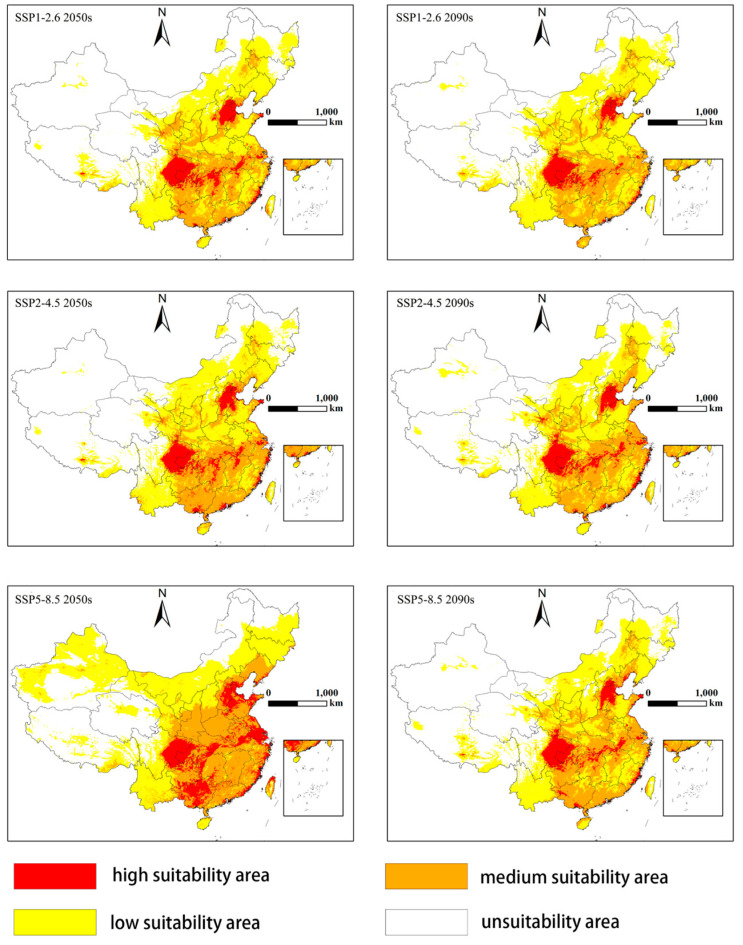
Potential distribution of *Sclerodermus sichuanensis* in the future (2050s, 2090s) under the SSP1-2.6, SSP2-4.5 and SSP5-8.5 climate change scenarios. The probability of *S. sichuanensis* is shown by the colour scale in the area. Red is a highly suitable area with a probability of higher than 0.66; Orange is a moderately suitable area with a probability of 0.33–0.66; Yellow is a poorly suitable area with a probability ranging from 0.05–0.33; White is an unsuitable area.

The Maxent model was used to predict the distribution of *S. sichuanensis* under SSP climate change scenarios of SSP1-2.6, SSP2-4.5, and SSP5-8.5, as shown in Figure 4. Compared with the current distribution, the high, medium, and low suitability areas predicted in the 2050s and the 2090s have changed to varying degrees. Among them, the projected area of all three suitable areas increases under the 2050s SSP5-8.5 scenario. The suitable region is expanding north and northwest of Inner Mongolia, Gansu, and Xinjiang. Compared with the current suitability area, the high suitability area is increasing in the 2050s under the SSP5-8.5 scenario. Under other SSP conditions, the area of high suitability is decreasing, especially in Jiangsu, Shanghai, Zhejiang, and other coastal areas.

Table 4 shows that according to the SSP1-2.6 scenario predicted by the model, the areas of high suitability and low suitability will decrease by 2050, with the reduced areas of 3616 km^2^ and 8158 km^2,^ respectively, accounting for 12.6% and 4.8% of the current predicted areas respectively. Only Jiangxi, Guangdong, and other places in the southeast of the moderate suitability area increased significantly, with an increase of 5571 km^2^ or 7.2% compared with the current area. By the 2090s, the growth rate of suitability change is different from that of the 2050s. The highly suitable areas in Jiangsu, Zhejiang, and other coastal areas have been largely reduced to moderately suitable areas. The area of high suitability decreased by 6237 km^2^, 21.7% compared with the current predicted area. The area of medium adaptability also decreased by 0.8%, while the area of low adaptability increased by 0.3%. Under the SSP2-4.5 scenario, the high suitability area and the low suitability area are decreasing when predicted at the 2050s and 2090s service life points and the medium suitability area is expanding to the southeast of Jiangxi, Guangdong, Fujian, and Zhejiang, with an increase of 6188 km^2^ (8.0%) in 2050s and 6773 km^2^ (8.7%) in the 2090s. Under the SSP5-8.5 scenario, the largest change in the prediction took place in 2050, with all kinds of suitability areas increasing. The high suitability area of *S. sichuanensis* increased by 15,714 km^2^, with an increase of 54.8%, the medium suitability area increased by 33,797 km^2^ (43.4%), and the low suitability area increased by 31,784 km^2^ (18.6%), with a total increase of 81,295 km^2^, accounting for 0.9% of the total land area; by the 2090s, only the area with medium suitability increased by 11,867 km^2^, with an increase of 15.25%, while the area with high suitability and the area with low suitability decreased by 14.9% and 7.7% respectively. From this point of view, the suitability area of *S. sichuanensis* will be developed in the 2050s, with the largest increase at SSP5-8.5.

### 3.5. Environmental Variables Affecting the Geographical Distribution 

**Figure 5:**
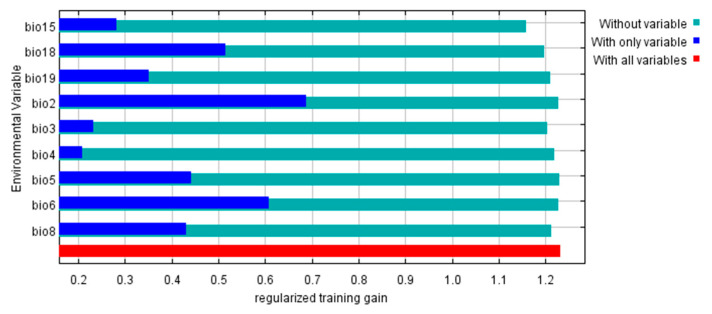
Importance of environmental variables to *Sclerodermus sichuanensis* by Jackknife test.

MaxEnt software was adopted to analyze the environmental variables that significantly impact the distribution of *S. sichuanensis* by the jackknife test method. As shown in Figure 5, “with only variable”, “without variable,” and “with all variables” are checked to show the importance of environmental variables to the distribution. The length of the blue band in the figure represents the importance of each variable to the species distribution when used alone. The more important the variable is to the species distribution, the longer the blue band will be displayed in the figure. The figure shows that nine environmental variables have different degrees of influence on the predicted distribution of *S. sichuanensis* in the future. Among these key environmental variables, bio2, bio6, and bio18 have regularized training gains above 0.4. The gain of bio2, which is close to 0.7, is the most critical environmental variable affecting the distribution of *S. sichuanensis*.

**Figure 6:**
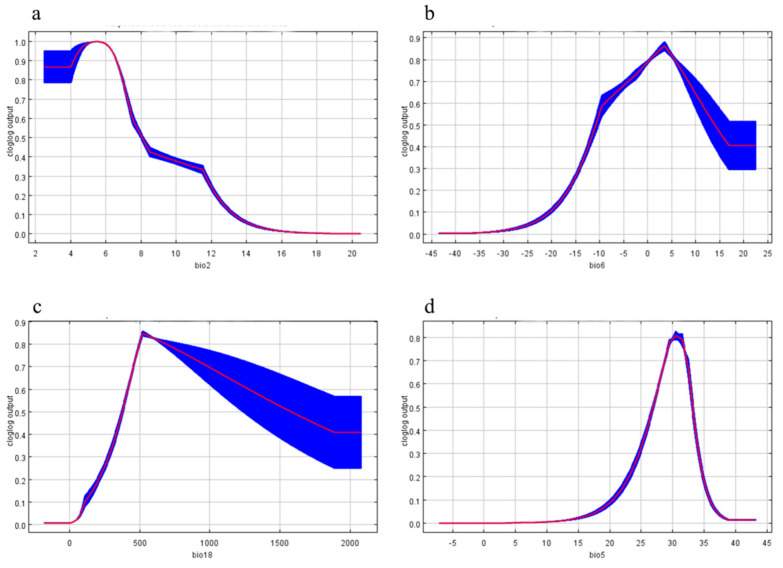
Response curves of the environmental variables that contributed most to the MaxEnt models. (**a**) Mean Diurnal Range (bio2). (**b**) Min Temperature of Coldest Month (bio6). (**c**) Precipitation of Warmest Quarter (bio18). (**d**) Max Temperature of Warmest Month (bio5).

Figure 6 shows the response curve of environmental variables to the distribution probability of *S. sichuanensis* in the MaxEnt model, which reflects the potential suitability change caused by changes in environmental variable values. The distribution value is divided according to the division method of evaluation probability in the IPCC report, and the threshold value is 0.5. As shown in the figure, each environmental variable has an optimal value for its impact on the suitability distribution. When the environmental variable is at its optimal value, the suitability distribution probability reaches the maximum. Conversely, when the environmental variable value is lower than or higher than the optimal value, the suitability distribution probability will decrease.

According to the response curve of environmental variables to the distribution probability of *S. sichuanensis* shown in Figure 6, the variable range of its potential distribution is determined. The four environmental variables that have the greatest impact on the potential distribution of *S. sichuanensis* are Mean Diurnal Range (bio2), Min Temperature of Coldest Month (bio6), Precipitation of Warmest Quarter (bio18), Max Temperature of Warmest Month (bio5). The appropriate range corresponding to each variable is shown in Table 5. The temperature variation range of the Mean Diurnal Range is 2.40–8.10 °C, and the optimal variation temperature is 5.40 °C. At this time, the predicted suitability value is 1.0. The suitability range of the Min Temperature of the Coldest Month is −11.00–13.50 °C. When the temperature of the curve is lower than −20 °C, the suitability value is lower than 0.1 and changes slowly with the temperature. When the temperature rises from −20 °C to −10 °C, the suitability change curve increases rapidly. When the temperature continues to rise to 3 °C, the optimum value is reached. When the temperature is higher than 3 °C, the suitability value decreases but remains above 0.4, indicating that *S. sichuanensis* is more suitable for living in warm environments than in the cold. The suitability range of the Precision of the Warmest Quarter is relatively wide. When the precipitation is between 381.66 mm and 1569.35 mm, the predicted suitability is greater than 0.5. When the precipitation is 0–500 mm, the curve is steep, and the prediction suitability increases rapidly. When the precipitation is 534.99 mm, the prediction suitability reaches the peak. Different from the previous curve, when the precipitation is greater than 534.99 mm, the curve is relatively gentle, and the prediction suitability decreases slowly, indicating that *S. sichuanensis* has a strong humidity tolerance. The temperature variation range of the Max Temperature of Warm Month is 26.69–33.04 °C, the suitability temperature variation range is small, and the curve reaches the peak when the Temperature is 30.41 °C. The prediction suitability is 0 when the temperature is below 13 °C, the curve rises sharply at 13–30.41 °C, and the curve declines more steeply at 30.41–40 °C.

## 4. Discussion

According to the established environmental variables and the existing distribution positions of species, using the MaxEnt model [26] established based on maximum entropy theory, the current geographical range of suitability distribution of *S. sichuanensis* was analyzed [27], and the suitability distribution changes and environmental adaptation of it under future climatic conditions were also predicted. Using the ROC curve and AUC value to evaluate the MaxEnt model, the results showed that the model has a good prediction effect on the distribution of *S. sichuanensis*.

Environmental variables such as temperature, precipitation, and altitude have direct and indirect effects on the survival of insects [28]. They are closely related to the individual development and population dynamics of insects. Among them, temperature and humidity directly affect insect growth, development, survival, and reproduction. In addition, the temperature in the environment affects the metabolic rate of insects. The combination of temperature and other factors will also indirectly affect the survival and development of insects [29,30]. In this study, we used the Jackknife test experiment to screen the important environmental variables that affect the distribution of *S. sichuanensis*, used the Pearson correlation coefficient to screen the variables again, and determined nine important variables from 19 environmental variables, bio15, bio18, bio19, bio2, bio3, bio4, bio5, bio6, bio8. Combined with the Jackknife test, it showed that the most influential variables were Mean Diurnal Range (bio2), Min Temperature of Coldest Month (bio6), Precipitation of Warmest Quarter (bio18), Max Temperature of Warmest Month (bio5).

Several essential variables indicate that temperature and precipitation significantly affect the geographical distribution of *S. sichuanensis*, and temperature factors (Mean Diurnal Range, Min Temperature of Coldest Month, and Max Temperature of Warmest Month) are more important than precipitation factors (Precision of Warmest Quarter). The optimal variable temperature of the Mean Diurnal Range is 5.40 °C, the optimal temperature in the coldest season is 3 °C, and the optimal temperature in the warmest season is 30.41 °C, which is consistent with the conclusion that Zhou et al. (2003) concluded that *S. sichuanensis* is a kind of insect that prefers high temperature. Besides, *S. sichuanensis* is a completely metamorphosed insect. Its individual development mainly goes through four stages: egg, larva, pupa, and adult. The duration of each development stage, from the egg to the adult stage, is gradually extended.

In contrast, the duration of the entire development cycle will shorten with the increase in temperature. Therefore, the warm temperature will reduce the development time and provide a new idea for rapidly breeding the parasitic wasps. Zhou et al. (2003) found that those parasitic wasps overwinter as adults in the borers of longicorn beetles and only started to go out when the temperature exceeded 20 °C in April to May of the following year to look for hosts [31], carrying out supplementary nutrition, oviposition parasitism, and child care management activities [32], which also showed that *S. sichuanensis* would put necessary life courses under warm temperatures. In the coldest season, when the temperature is −20–3 °C, *S. sichuanensis* also shows good adaptability, indicating that it also has strong adaptability to low temperatures. Research by Zhou et al. (2016) showed that *S. sichuanensis* can still recover its normal vitality after being treated at −18 °C for a short time for one minute or gradually cooled at −8 °C for one week. Zhou (2007) and Zhao (2020) also found that this species had cold solid tolerance in the study of low-temperature cultivation, and its cold tolerance mechanism was preliminarily studied. All above these results are consistent with this study.

Furthermore, the best precipitation in the warmest season is 534.99 mm, and the predicted suitability curve decreased slowly when the precipitation was greater than 534.99 mm, speculating that *S. sichuanensis* may have a strong adaptation to precipitation. Li et al. (2021) place the parasitic wasp on paper or in a container and flood them with water [33]. The wasp can quickly escape from the water or float on the water and climb out along the container wall. It can be seen that the parasitic wasp has strong water resistance. In the bore way harmed by the host, the parasitism rate will reduce when there is too much water (in the form of water immersion), which will even cause the death of the parasitic wasp. In humid environments, the larvae of the wasp are also vulnerable to the infection of metarhizium and cannot complete their development [34,35].

The simulation results of the Maxent model show that the highly suitable areas for *S. sichuanensis* in China are mainly in plains and basins areas such as Sichuan, Chongqing, Guizhou, Hebei, and Jiangsu, which are concentrated in southwest China and North China; the moderately suitable areas are concentrated in South China and Central China such as Guangxi, Hunan, and Hubei; and the most suitable areas for high and middle schools are subtropical monsoon climate, followed by a temperate monsoon climate. In the subtropical monsoon climate zone, it is hot and rainy in the summer and mild and rainy in the winter. The annual temperature is between 13 °C and 20 °C, and the average annual precipitation is 800 mm~1500 mm. These are consistent with the previous study on the impact of environmental factors on the distribution of *S. sichuanensis*. On the other hand, the temperate monsoon climate is hot and rainy in the summer and cold and dry in the winter, consistent with the above-mentioned *S. sichuanensis* prefers high temperatures and has strong cold resistance.

Under the three scenarios of SSP climate change with different degrees, the distribution of *S. sichuanensis* is quite different. Only in the high emission scenario (SSP5-8.5) the predicted high suitability area, medium suitability area, and low suitability area of *S. sichuanensis* will increase by 54.77%, 43.42%, and 18.64%, respectively, in the 2050s, with a total increase of 81,295 km^2^. In other SSPs, the area of high suitability areas predicted in the 2050s and the 2090s is decreasing, and only the areas of medium suitability areas and low suitability areas have a small increase or decrease. The growth scope of the suitable areas is expanding to Inner Mongolia, Gansu, Xinjiang, and other places. These areas are mainly located in North China and Northwest China. The climate belongs to the temperate continental climate. It is cold in the winter and hot in the summer. The annual and daily temperature ranges are large. The significant expansion of the suitable area may be related to the climate warming caused by SSP5-8.5, a high carbon dioxide greenhouse gas concentration emission. Climate warming has increased the temperature of Inner Mongolia, Xinjiang, and other places that should be in cold regions, making *S. sichuanensis* with strong cold tolerance dig out new territory.

The Maxent model used in this study has good performance among many prediction models [36], accurate prediction, simple operation, robust performance, and other advantages, but the use of the model is still not perfect [37]. The variables used in this study only involve temperature, rainfall and altitude, and other environmental impacts factors such as wind speed, airflow, air, and landform are not fully included in the model. The distribution area of *S. sichuanensis* hosts is also an important factor influencing its distribution. All environmental factors in the ecosystem are relatively restrained and affected. In addition to climate, geography, and other environmental factors, the biological characteristics of *S. sichuanensis* are all factors affecting distribution, such as nutrition supplements [38], storage density, and host type. There are many host species of *S. sichuanensis*, which has a good control effect on many kinds of longicorn beetles and is a great helper for pest control in forestry [39]. In future research, more influencing factors should be considered to improve the accuracy of predictions and provide more abundant reference information for using *S. sichuanensis*.

## 5. Conclusions

In this report, the data collected on the occurrence of *S. sichuanensis* and environmental factors were substituted into the Maxent model, and the data were extracted and analyzed by ArcGIS software. As a result, the current and future suitable habitat distribution of *S. sichuanensis* in China was obtained. The results showed that the important environmental variables affecting the distribution of *S. sichuanensis* were Mean Diurnal Range (bio2), Min Temperature of Coldest Month (bio6), Precision of Warmest Quarter (bio18), Max Temperature of Warmest Month (bio5). Currently, the high suitability area is mainly in southwest China and part of North China, while the medium suitability area is concentrated in South China and Central China. Under the SSP5-8.5 climate scenario, the predicted suitability area in the 2050s (2041–2060) will expand significantly to North China and Northwest China. This study explains the distribution of *S. sichuanensis* and environmental impact factors from a new perspective. It provides a reference for the ecological characteristics of *S. sichuanensis* and an essential reference for its use in forestry control.

## Figures and Tables

**Table 1:** Environmental variables used for distribution prediction of *Sclerodermus sichuanensis*.

Environmental Variables	Abbreviation
Annual Mean Temperature	bio1
Mean Diurnal Range (Mean of monthly [max temp–min temp])	bio2
Isothermality (bio 2/bio 7) (×100)	bio3
Temperature Seasonality (SD × 100)	bio4
Max Temperature of Warmest Month	bio5
Min Temperature of Coldest Month	bio6
Temperature Annual Range (bio5–bio6)	bio7
Mean Temperature of Wettest Quarter	bio8
Mean Temperature of Driest Quarter	bio9
Mean Temperature of Warmest Quarter	bio10
Mean Temperature of Coldest Quarter	bio11
Annual Precipitation	bio12
Precipitation of Wettest Month	bio13
Precipitation of Driest Month	bio14
Precipitation Seasonality (Coefficient of Variation)	bio15
Precipitation of Wettest Quarter	bio16
Precipitation of Driest Quarter	bio17
Precipitation of Warmest Quarter	bio18
Precipitation of Coldest Quarter	bio19
Altitude	Alt

**Table 2:** Percent contribution and the permutation importance of environmental variables affecting the distribution of *Sclerodermus sichuanensis*.

Environmental Variables	Abbreviation	Percent Contribution/%	Permutation Importance/%
Precipitation of Warmest Quarter	bio18	47.7	7.5
Temperature Seasonality (SD × 100)	Bio4	17.7	1.8
Precipitation Seasonality (Coefficient of Variation)	Bio15	7.6	0.6
Precipitation of Wettest Month	bio13	5.4	5.1
Isothermality (bio 2/bio 7) (×100)	Bio3	4.4	21.4
Min Temperature of Coldest Month	Bio6	4.1	0.8
Precipitation of Coldest Quarter	Bio19	3.5	4.7
Mean Diurnal Range (Mean of monthly [max temp–min temp])	Bio2	2.2	2.1
Max Temperature of Warmest Month	Bio5	1.6	2.2
Temperature Annual Range (bio5–bio6)	Bio7	1.1	0
Mean Temperature of Coldest Quarter	Bio11	1	24.5
Mean Temperature of Wettest Quarter	Bio8	1	0.2

**Table 3:** Predicted suitability for *Sclerodermus sichuanensis* in China under current climatic conditions.

Province	High Suitable Area (km^2^)	Medium Suitable Area (km^2^)	Low Suitable Area (km^2^)	No Suitable Area (km^2^)	Total Area of Provinces (km^2^)	Percentage of High Suitable Areas in Province (%)	Percentage of High Suitable Areas in China (%)
Sichuan	7082	1369	7788	9989	26,228	27.0	24.7
Chongqing	3374	534	548	0	4456	75.7	11.8
Guizhou	3237	5178	779	0	9194	35.2	11.3
Hebei	2836	4275	3919	281	11,311	25.1	9.9
Jiangsu	1782	3274	558	0	5614	31.7	6.2
Zhejiang	1586	2385	1477	0	5448	29.1	5.5
Guangxi	1518	10,013	541	0	12,072	12.6	5.3
Hunan	1054	7989	2125	0	11,168	9.4	3.7
Hubei	1030	6606	2479	0	10,115	10.2	3.6
Shandong	781	3176	4910	0	8867	8.8	2.7
Tianjin	699	5	0	0	704	99.3	2.4
Guangdong	668	4641	3681	0	8990	7.4	2.3
Beijing	656	307	29	0	992	66.1	2.3
Fujian	560	1503	4268	0	6331	8.9	2.0
Liaoning	407	3260	5080	277	9024	4.5	1.4
Shanghai	301	37	0	0	338	89.1	1.1
Anhui	269	5004	2424	0	7697	3.5	0.9
Yunnan	233	1054	17,770	689	19,746	1.2	0.8
Xizang	191	440	7708	57,510	65,849	0.3	0.7
Gansu	79	1479	9570	12,793	23,921	0.3	0.3
Inner Mongolia	77	3276	35,634	35,383	74,370	0.1	0.3
Shaanxi	54	2685	8960	41	11,740	0.5	0.2
Hong Kong	52	0	0	0	52	100.0	0.2
Jiangxi	41	1975	6780	0	8796	0.5	0.1
Taiwan	35	507	975	320	1837	1.9	0.1
Hainan	18	874	786	0	1678	1.1	0.1
Henan	11	2048	7231	0	9290	0.1	0.0
Jilin	8	2065	6598	3592	12,263	0.1	0.0
Shanxi	1	682	7942	566	9191	0.0	0.0
Xinjiang	0	23	4680	96,455	101,158	0.0	0.0
Qinghai	0	61	2585	38,446	41,092	0.0	0.0
Heilongjiang	0	186	10,586	20,588	31,360	0.0	0.0
Ningxia	0	930	2104	0	3034	0.0	0.0
China	28,640	77,841	170,515	276,930	—	0.3	—

**Table 4:** Predicted suitable areas for *Sclerodermus sichuanensis* under current and future climatic conditions.

		Predicted Area (km^2^)			Comparison with Current Distribution (%)		
Decade	Scenarios	High Suitable	Medium Suitable	Low Suitable	High Suitable	Medium Suitable	Low Suitable
current	—	28,691	77,841	170,515	—	—	—
2050s	SSP1-2.6	25,075	83,412	162,357	−12.6	7.2	−4.8
	SSP2-4.5	25,767	84,029	158,285	−10.2	8.0	−7.2
	SSP5-8.5	44,405	111,638	202,299	54.8	43.4	18.6
2090s	SSP1-2.6	22,454	77,184	170,955	−21.7	−0.8	0.3
	SSP2-4.5	26,215	84,614	156,550	−8.6	8.7	−8.2
	SSP5-8.5	24,423	89,708	157,467	−14.9	15.3	−7.7

**Table 5:** A suitable range of environmental variables for the potential distribution of *Sclerodermus sichuanensis*.

Environmental Variables	Unit	Suitable Range	Optimum Value
bio2	°C	2.40–8.10	5.40
bio6	°C	−11.00–13.50	3.00
bio18	mm	381.66–1569.35	534.99
bio5	°C	26.69–33.04	30.41

## Data Availability

The data supporting the results are available in a public repository at: Qianqian Qian (2023): Locations of *Sclerodermus sichuanensis*. figshare. Dataset. https://doi.org/10.6084/m9.figshare.22794062.v2, accessed on 11 May 2023.
